# Characteristics and Kinetics of Rosin Pentaerythritol Ester via Oxidation Process under Ultraviolet Irradiation

**DOI:** 10.3390/molecules23112816

**Published:** 2018-10-30

**Authors:** Yuanlin Li, Xiongmin Liu, Qiang Zhang, Bo Wang, Chang Yu, Haroon Ur Rashid, Yiming Xu, Li Ma, Fang Lai

**Affiliations:** School of Chemistry and Chemical Engineering, Guangxi University, Nanning 530004, China; lylgzgy@163.com (Y.L.); wwwbxp@126.com (Q.Z.); jianbowang04@163.com (B.W.); 13036819261@163.com (C.Y.); haroongold@gmail.com (H.U.R.); xuyimingcorey@163.com (Y.X.); gxumali@126.com (L.M.); laifang200609@126.com (F.L.)

**Keywords:** rosin pentaerythritol ester, photo-oxidation, kinetics, UV

## Abstract

A self-designed reaction device was used as a promising equipment to investigate the oxidation characteristics and kinetics of rosin pentaerythritol ester (RPE) under UV irradiation. Photo-oxidation kinetics and the initial quantum yield (Φ) of RPE were calculated. The initial oxidation product of the photo-oxidation reaction—peroxide was analyzed by iodimetry. The peroxide concentration is related to the light intensity (I) and the temperature (T), and the increasing T and I would destabilize the RPE by accelerating peroxide forming. Photo-oxidation of RPE follows the pseudo first-order reaction kinetics. The relationship between activation energy and logarithm of light intensity (ln I) is linear, and it is expressed as Ea = −4.937ln I + 45.565. Φ was calculated by the photo-oxidation kinetics, and the average value of Φ was 7.19% in the light intensity range of 200–800 μW cm^−2^. This research can provide fundamental information for application of RPE, and help obtain a better understanding of the stability of rosin esters.

## 1. Introduction

Rosin is a significant and renewable natural product produced by the resin of some conifers, such as pine trees. It is a non-volatile transparent or translucent solid and plays an important role in daily life. The main components of rosin include abietic acid and a few other resin acids [1]. The carboxylic group of rosin can be coupled to different alcohols such as pentaerythritol, methanol, ethylene-glycol, and glycerol via esterification, and rosin esters are some of the most common rosin derivatives [2,3]. China’s annual production of gum rosin is about 400,000 tons, accounting for 60% of the world’s total production. In 2017, the output of rosin ester products in China was about 100,000 tons [4].

**Figure 1:**
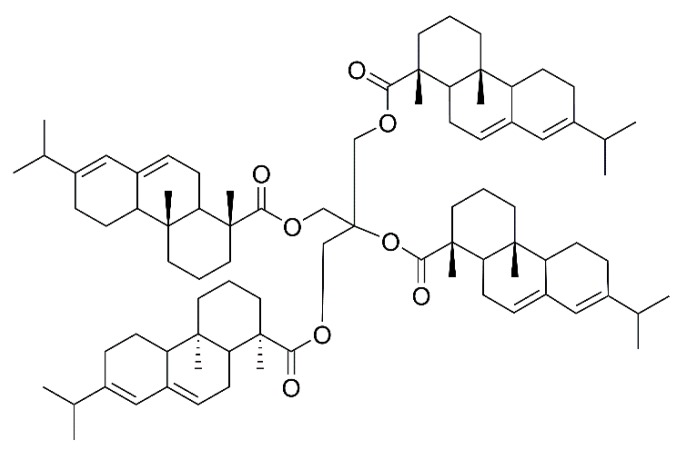
The molecular structure of RPE.

Rosin pentaerythritol ester (RPE) is a common modified rosin product obtained by esterification of rosin acid and pentaerythritol, and its molecular structure is shown in Figure 1. RPE is widely used in paper, food, inks and coatings, as well as in adhesive industries [5,6,7,8,9]. Lee et al. [10] used RPE as an additive to polyvinylidene fluoride binders and it effectively improved the lithium-titanium oxide (LTO) electrochemical performance. Kumooka et al. [11] reported the addition of the rosin pentaerythritol ester, causing improvement in the cohesive strength of the adhesive. Sangjun et al. [12] prepared a pressure-sensitive adhesive (PSA) using RPE as tackifier, which promised sustainability and high adhesion properties.

However, concerns about the stability of RPE do exist due to the presence of active conjugated double bonds in RPE molecular structure. Light, heat and oxygen in air destabilize RPE upon contact, which may cause its color degradation and limit its applications. Ploeger et al. [13] studied the light stability of adhesives used as consolidants for flaking or fragile paint layers on furniture, paintings, polychrome sculpture and other cultural objects. It was observed that the consolidants could show yellowing and shifts in solubility due to the presence of RPE. Narayanan et al. [14] investigated the light transmitted through films in both UV and visible regions, and rosin based biocomposite films exhibited distinct light absorption feature. Pacholski et al. [15] successfully proved that the oxidation of rosin ester tackifier led to significant tack loss by XPS and adhesion testing.

Several investigations regarding the oxidation of rosin acids have been performed. Prinz et al. [16] identified six oxidation products of rosin methyl ester and pointed out that the oxidation sites in rosin methyl ester are the conjugated double bonds. Qin et al. [17,18] reported the thermal and UV-induced oxidation kinetics of rosin and collected the kinetic data. Ren et al. [19] found that thermal oxidation process of rosin is two-step reaction: (a) rosin peroxides are formed; (b) the peroxides are then cracked to produce carbonyl-containing products. However, there are only a few reports about the stability of RPE. In particular, the influence of UV irradiation on its oxidative characteristics and stability has never been investigated. Due to its low cost and convenience, UV lamp is large-scale used in adhesive curing and daily disinfection. The π-electronic conjugated alkene structures of RPE are related to the photoactive properties. The potential effects of RPE oxidation under UV irradiation must be carefully examined.

The purpose of this study was to investigate the oxidative stability and reaction process of RPE under UV irradiation and is mainly focused on the oxidation kinetics and formation of RPE peroxide. The photo-oxidation experiments were performed on a self-designed gas-solid photoreactor. The activation energy (Ea), photo quantum yield (Φ) and parameters of transition state were calculated from the photo-oxidation studies. Furthermore, the peroxide formation during photo-oxidation was detected by iodimetry. This research could enhance our understanding of stability and characteristics of RPE oxidation. The results may be quite instructive for avoiding the problems of RPE discoloration, unacceptable tack and peel issues during its production, storage, transportation, and usage. Moreover, this study also provides a theoretical basis to predict the stability of other rosin products.

## 2. Results and Discussions

### 2.1. UV Spectra Characteristics of RPE

**Figure 2:**
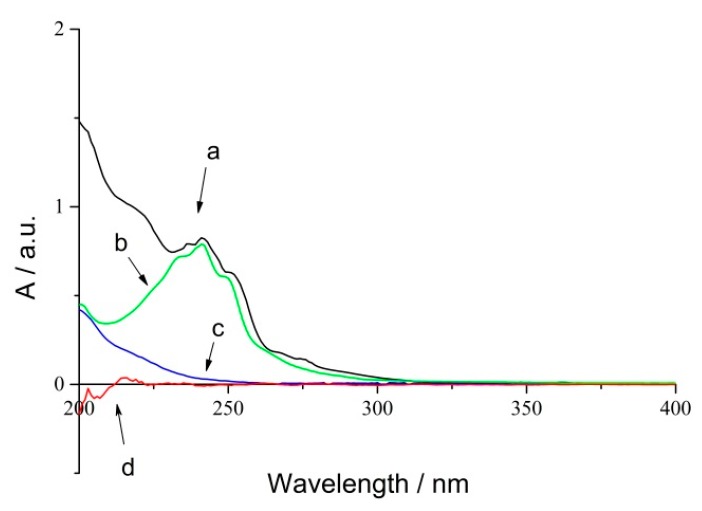
UV spectra of RPE photo-oxidation process (**a**) RPE; (**b**) Rosin; (**c**) Oxide of RPE; (**d**) Blank PE film.

RPE showed significant absorption in UV spectroscopy because of the conjugated group. Herein, a UV radiometer was used to detect the changes of conjugated bonds during the photo-oxidation process. The UV absorption spectra of RPE (a), rosin (b), oxide of RPE (c) and polyethylene film (d) are shown in Figure 2.

Curve (a) and (b) revealed that RPE and rosin have the same absorbance peaks located at 242 nm, and curve (d) represents blank polyethylene film. Thus, an absorbance of 242 nm could be used to study the photo-oxidation of RPE. Curve (c) suggested that the absorption of oxide was noticeable even though it showed a featureless absorbance peak within the same range. An iterative calculation method was used to obtain the kinetic data.

### 2.2. RPE Oxidation Characteristics Under UV Irradiation

**Figure 3:**
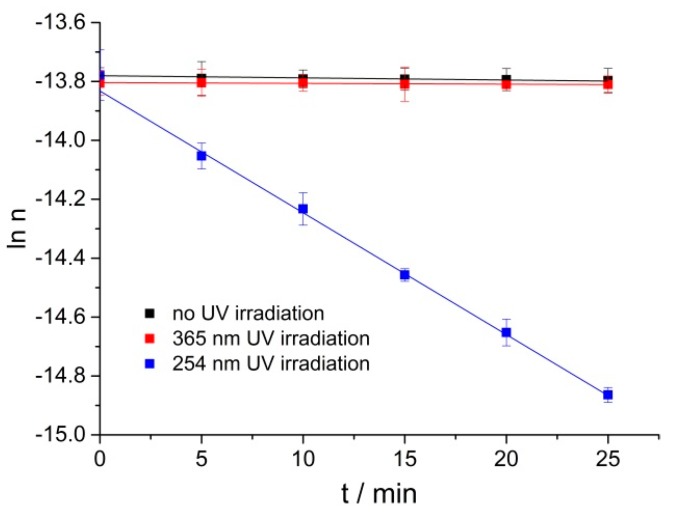
The change of natural logarithm of RPE moles with time under no UV irradiation, 365 nm and 254 nm UV irradiation at 40 °C.

254 nm UV lamps play an important role in our daily lives and are widely used in adhesive curing and disinfection [20]; more than 96.5% of the solar UV radiation which reaches the earth’s surface is UVA (365 nm central wavelength) radiation [21]. Liu et al. [22] reported gum rosin could get oxidized under 365 nm radiation. The time-dependent oxidations of RPE under no UV irradiation, 365 nm and 254 nm UV irradiation at 40 °C were compared. As shown in Figure 3 under no or 365 nm UV irradiation, RPE could hardly be oxidized at 40 °C; whereas, RPE could be oxidized efficiently under 254 nm UV irradiation. It is reported that the photo-oxidation process follows a pseudo first-order kinetic model. Yuri et al. [23] found that the photo-oxidation process of Δ^9^-tetrahydrocannabinol under UV irradiation followed pseudo first-order kinetics, Pamela et al. [24] studied the photo-oxidation of bromoxynil and trifluralin, and the photochemical rate followed first-order kinetics. The RPE oxidation under 254 nm UV is well fitted by pseudo first-order kinetics. Herein, it is necessary to study the RPE photo-oxidation process under 254 nm UV irradiation.

### 2.3. Peroxide of RPE by Iodimetry

**Figure 4:**
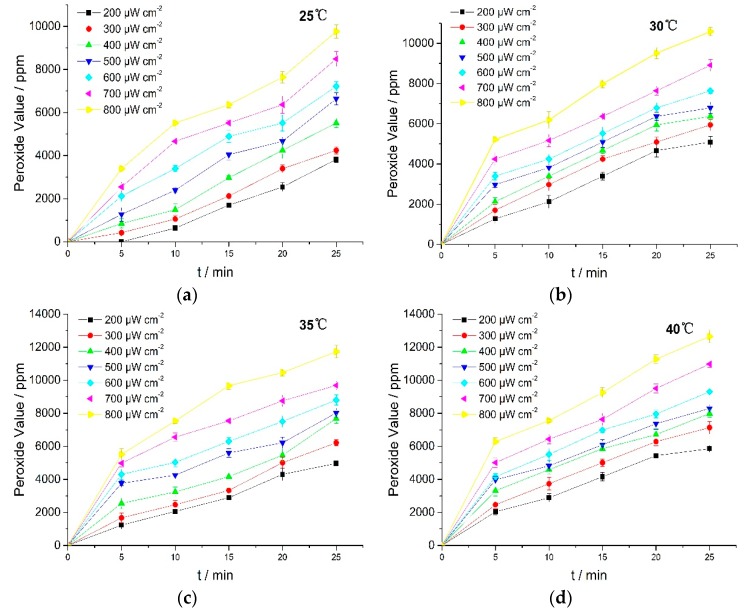
PV of RPE at various temperatures. (**a**) 25 °C; (**b**) 30 °C; (**c**) 35 °C; (**d**) 40 °C.

The UV irradiation results showed that the photo-oxidation process occurred through oxygen absorption, followed by possible peroxide formation. To obtain the evidence about peroxide formation, the peroxide value (PV) was measured by iodimetry. Results are shown in Figure 4.

Figure 4 showed that PV rose with the increasing in light intensity and reaction temperature. These results revealed that UV irradiation induced oxidation of RPE which then led to the formation of more peroxides. The unsaturated bonds in RPE molecular structures were oxidized to form peroxides under UV irradiation, and the formation rates of peroxides and free radicals (R) during the reaction were critical to the photo-oxidation [25]. Peroxide formation thus contributed toward potent off-flavors and had a direct impact on the quality of many RPE products. Therefore, it is necessary to prevent RPE from exposure to UV light or add in light stabilizers in RPE usage.

### 2.4. Kinetics of RPE Oxidation Under UV Irradiation

**Figure 5:**
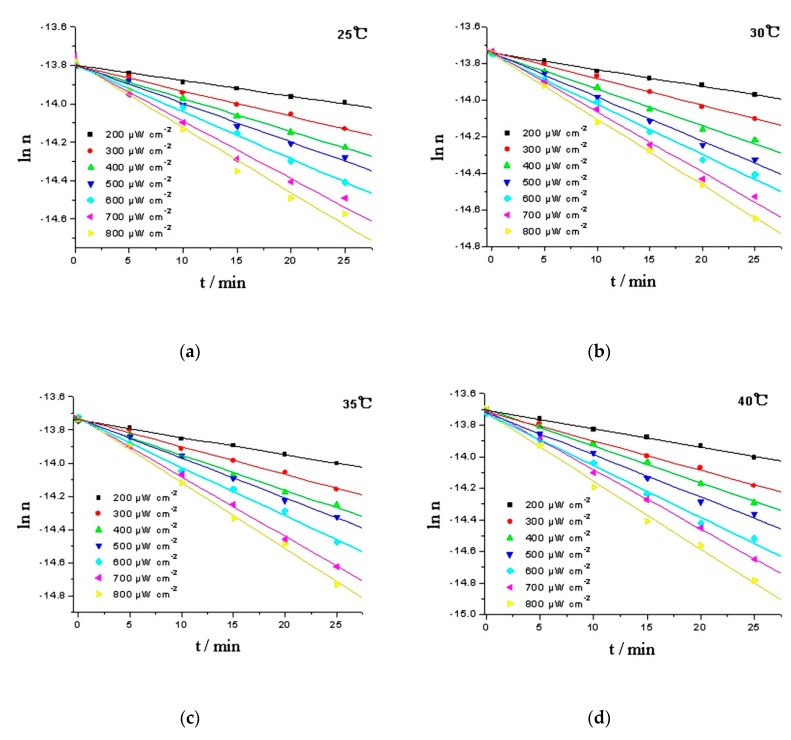
Comparison of the experimental (data point) and calculated values (line) of RPE photo-oxidation at various temperatures. (**a**) 25 °C; (**b**) 30 °C; (**c**) 35 °C; (**d**) 40 °C.

The logarithm of moles (ln n) was plotted against time (t), and the moles of RPE was calculated from the working curve of RPE, y = 1.309 × 10^3^x − 0.363, and oxide of RPE, y = 16.654 × 10^3^x + 0.0256. The linear response of ln n to time suggests that the oxidation of RPE is a pseudo first-order reaction. Intensity of the irradiation is an important factor that influencing the photoactivity of organic chemicals [26]. In daily disinfection, the intensity at one meter under 254 nm UV lamp should be above 180 μW cm^−2^ [27]. Hence, the kinetics of the RPE photo-oxidation process were studied in a temperature range of 25–40 °C and a light intensity range of 200–800 μW cm^−2^ (Figure 5). Using the Levenberg-Marquardt method and employing the Matlab software package to calculate the rate constants, and the activation energies (Ea) can be estimated using the Arrhenius equation (Equation (1)) [28]. The results are given in Table 1.

k = A exp(−Ea/RT)(1)

Table 1 showed that the reaction rate constants increased with the rising temperature and light intensity. The enhanced effect of the reaction rate constants could be attributed to reactive radicals formation by UV irradiation and absorbed oxygen to form peroxides [29]. The calculated apparent activation energies were 12.60–19.17 kJ mol^−1^, and the activation energies of RPE photo-oxidation reduced by increasing UV irradiation, indicating that UV irradiation could accelerate the oxidation rate of RPE. In addition, Ea and the logarithm of light intensity have a linear relationship, and it is expressed as:

Ea = −4.937ln I + 45.565(2)

A good fit of the kinetic parameters would confirm the suitability of the mathematical model to describe the reaction [30,31]. An F-test was performed to assess the accuracy of the model results when F > F (m, N-m, 0.95), which denotes the 95% value of the Fisher distribution with (m, N-m) degrees of freedom. In our case, m was the number of model parameters, 3; and N was the number of sampling points, 6. Reasonable confidence intervals of the estimated parameters could be seen in Table 2, and the results of statistical tests are shown in Table 2. It is obvious that all of the correlation coefficients (R^2^) are larger than 0.99, the sums of errors (Q) are small (1.26 × 10^−4^~6.26 × 10^−3^) and the calculated F-values are greater than the F_t_-value (F_0.05_ (3, 3) = 3.93) multiplied by 10, indicating that the kinetic model is significant at 95% confidence level.

### 2.5. The Initial Quantum Yield

The quantum yield (Φ) is one of the fundamental parameters for a photochemical reaction. It reflects the efficiency of a photo-chemical reaction. The results are presented in Table 3.

Based on Equations (8) and (9), the quantum yield was calculated at 25 °C under the wavelength of 254 nm. Φ does not vary with variation in UV light intensity after the initial reaction phase: it is 7.19% on average. It indicates that the larger steric hindrance caused by esterification of rosin retarded the absorption of photon, but the influence of 254 nm irradiation should not be ignored, and it required some improvement through addition of adequate amounts of antioxidants when producing RPE.

### 2.6. Transition State Activation Energy

Regarding photo-oxidation kinetics of RPE, calculation of transition state parameters may also provide valuable information [32]. The formation of activation complex is considered to follow the transition state theory, for example, the magnitude of the activation enthalpy (∆H^≠^) and activation entropy (∆S^≠^) could indicate the transitional state of the reaction [33]. The activation free energy (∆G^≠^), the activation enthalpy (∆H^≠^) and the activation entropy (∆S^≠^) at all temperatures evaluated under UV irradiation are presented in Table 4.

From Table 4, thermodynamics properties of the formation of the activated complex were obtained. ∆G^≠^ represents the difference between the transitional state and reactants [34], and its values ranged from 81.367 to 88.574 kJ mol^−1^. ∆G^≠^ was used to determine the spontaneity of RPE photo-oxidation process at all temperatures tested. The positive sign means that photo-oxidation is an endergonic reaction and not spontaneous [35]. ∆H^≠^ measures the energy barrier that the reacting molecules must overcome and is related to the strength of the bonds broken and made in the formation of the reactant molecules to the transition state [36]. ∆H^≠^ values were similar under each set of conditions evaluated in this study, varying between 10.002 and 16.695 kJ mol^−1^. The positive sign of ∆H^≠^ represents an endothermic state between reactants and activated complex, and heat input is required to bring the RPE to the transition state so as to get oxidized [37,38]. Moreover, ∆H^≠^ becomes lower with the light intensity increases; it indicates that UV irradiation could induce the formation of activated complex. ∆S^≠^ is a measure of changes in the disorder of molecules in the system, and the negative value of ∆S^≠^ shows that the degree of disorder of transition state was lower as compared to reactants in the ground state [31]. 

## 3. Materials and Methods

### 3.1. Chemicals

RPE was obtained from Guangxi Wuzhou Pine Chemicals Ltd. (Guangxi, China). Acetone (purity > 99%), acetic acid (purity > 99%), sodium thiosulfate (purity > 99%), chloroform (purity > 99%) and potassium iodide (purity > 99%) were purchased from Xilong Chemical Co., Ltd. (Guangxi, China). Soluble starch (AR) was purchased from Guangdong Guanghua Sci-Tech Co., Ltd., (Guangdong, China). 

### 3.2. Design of Photo-Oxidation Equipment

**Figure 6:**
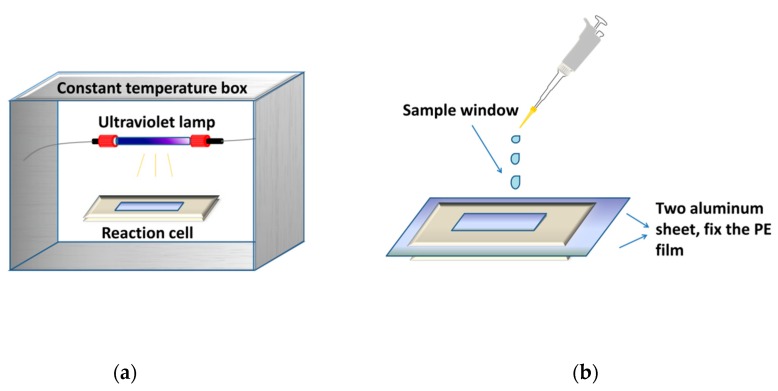
The gas-solid photoreactor used to investigate photo-oxidation process. (**a**) photo-oxidation equipment; (**b**) structure of reaction cell.

Figure 6 showed a gas-solid photoreactor used in this study. The photo-oxidation reaction of RPE was conducted via polyethylene film, fixed by two aluminum sheets.

A low-pressure (LP) UV lamp (Philips, TUV G6T5, 6 W, 254 nm) was placed over the photoreactor. The film area of the photoreactor is 3.0 × 1.7 cm^2^. The surface irradiance was measured to be 200 μW cm^−2^ to 800 μW cm^−2^ by a UV-C ultraviolet radiometer (Shanghai Baoshan Gucun Optic Instrument Factory, Shanghai, China). The surface irradiance was adjusted by changing the distance between the sample and the UV lamp.

### 3.3. Photo-Oxidation

All experiments were carried out in the photoreactor described above. RPE (0.35 g) was dissolved in acetone (5 mL). Then, 20 μL of as-prepared solution was measured and added dropwise to photoreactor. After the solvent was fully evaporated in vacuum at 20 °C, the sample formed a thin film with a film area of 5.1 cm^2^. The testing sample was exposed to the UV irradiance beam, and the photo-oxidation of RPE was conducted by UV on its film surface. The kinetic runs were then performed at 25 °C to 40 °C. The photoreactor (as a reactor and a sample cell) was then placed into the UV spectrophotometer (Agilent-8453E, Agilent Technologies Co., Ltd., Santa Clara, CA, USA) every 5 min for quantitative analysis of RPE by external standard method.

### 3.4. Peroxide Analysis by Iodimetry

Peroxide value (PV) of photo-oxidation was determined via iodimetry. Sample on the film of photoreactor was dissolved in chloroform and added to aqueous potassium iodide. Mill equivalents of peroxide were measured by titration with sodium thiosulfate solution [39].

2KI + ROOH + H_2_O = I_2_ + 2KOH + ROH (3)

I_2_ + 2Na_2_S_2_O_3_ = Na_2_S_4_O_6_ + 2NaI (4)

### 3.5. Calculation Method of Oxidation Kinetics

Rate of chemical reaction (r) of RPE photo-oxidation can be expressed in Equation (5).

r = −dn_A_/dt = k_1_n_A_^α^n_B_^β^ (5)

where k_1_ represents the first-order rate constant of RPE photo-oxidation; n_A_, n_B_ represent the reaction molarity of RPE and O_2_ respectively; α denotes the photo-oxidation order of RPE; β shows the photo-oxidation order of O_2_. Considering the excess air, the photo-oxidation process could be expressed in Equation (6), where n_B_^β^ is a constant.

r = −dn_A_/dt = kn_A_, k = k_1_n_B_^β^ (6)

Equation (6) can also be expressed by integration as follows:

ln n_A_ = −kt + ln n_A,0_ (7)

The kinetic rate constant can be calculated by the slope of the logarithm of moles (ln n_A_) vs. Time (t).

### 3.6. Calculation Method of Quantum Yield

Quantum yield (Φ) is the ratio of the amount of reactant divided by the amount of absorbed photons (typically for monochromatic radiation absorbed by the reactant), according to the IUPAC definition [40]. The number of moles of photons absorbed is

R(t) = (I_0_ − I_1_)S/(N_0_hυV) (8)

where S is the reaction area (5.1 cm^2^), I_0_ and I_1_ denote the incident and emergent light intensity (μW cm^−2^) respectively, N_0_ is Avogadro’s number (6.023 × 10^23^), V is the volume of RPE (6.5 cm^3^), h is Planck’s constant (6.63 × 10^−34^ J s), and υ is the frequency of UV light (254 nm). Therefore,

Φ = (−dn_A_/dt)/R(t) = −kn_A_/R(t), (9)

where t indicates the oxidation time (min), −dn_A_/dt is the oxidation rate of RPE (mol dm^−3^ min^−1^), k shows the overall rate constant (min^−1^), n_A_ is the number of moles of RPE which has reacted [41].

### 3.7. Estimation Method of Transition State Thermodynamics

The activation enthalpy (∆H^≠^) and the free energy of inactivation (∆G^≠^) at each temperature were obtained using Equations (10) and (11), respectively [32,42]:

∆H^≠^ = Ea − RT (10)

∆G^≠^ = −RTln[(k ∙ h)/(k_B_T)] (11)

where k_B_ (1.3806 × 10^−23^ J K^−1^) is the Boltzmann’s constant.

From Equations (10) and (11), it is possible to calculate the activation entropy (∆S^≠^):

∆S^≠^ = (∆H^≠^ − ∆G^≠^)/T (12)

## 4. Conclusions

Characteristics and kinetics of RPE oxidation process under UV irradiation were investigated with the employment of a self-designed gas-solid photoreactor. This is a promising experimental method to make experimental conditions close to the actual application situation. Based on this study, the main conclusions are as follows:

(1) In air at room temperature, RPE could be oxidized under 254 nm UV irradiation, while no oxidation was observed under no or 365 nm UV irradiation.
(2) RPE could form a high level of peroxides in its photo-oxidation process. Light intensity (I) and temperature (T) are associated with the peroxide concentration. The increasing T and I would destabilize the RPE by accelerating peroxide formation.
(3) The photo-oxidation process of RPE followed pseudo first-order kinetics, Ea and the logarithm of light intensity have a linear relationship: Ea = −4.937ln I + 45.565. In the light intensity range of 200–800 μW cm^−2^, the average value of initial photo-oxidation quantum yield was calculated to be 7.19%.
(4) The parameters of the transition state demonstrated that the photo-oxidation of RPE was an endothermic, non-spontaneous and ordered process.

## Figures and Tables

**Table 1:** Kinetic parameters for the photo-oxidation of RPE.

I (μW cm^−2^)	k (min^−1^)				Ea (kJ mol^−1^)	R^2^
	25 °C	30 °C	35 °C	40 °C		
200	0.00815 ± 0.0005	0.00910 ± 0.0002	0.01042 ± 0.0003	0.01076 ± 0.0001	19.17	0.998
300	0.01317 ± 0.0001	0.01462 ± 0.0011	0.01674 ± 0.0007	0.01858 ± 0.0003	18.12	0.997
400	0.01714 ± 0.0003	0.01965 ± 0.0003	0.02123 ± 0.0006	0.02336 ± 0.0012	15.63	0.988
500	0.02005 ± 0.0016	0.02248 ± 0.0010	0.02403 ± 0.0003	0.02675 ± 0.0006	14.47	0.991
600	0.02440 ± 0.0002	0.02730 ± 0.0007	0.02906 ± 0.0004	0.03237 ± 0.0002	14.13	0.987
700	0.03002 ± 0.0011	0.03287 ± 0.0004	0.03582 ± 0.0015	0.03861 ± 0.0005	13.30	0.999
800	0.03386 ± 0.0002	0.03586 ± 0.0002	0.03962 ± 0.0007	0.04301 ± 0.0010	12.60	0.992

**Table 2:** Results of statistical tests of the proposed model for the photo-oxidation of RPE.

Temperature (°C)	R^2^	Q	F
25	0.992	6.26 × 10^−3^	494.76
30	0.997	4.43 × 10^−3^	1239.60
35	0.999	1.26 × 10^−4^	252.40
40	0.999	1.67 × 10^−4^	243.70

**Table 3:** The effect of varying the light intensity on the quantum yield (Φ) of RPE during its photo-oxidation.

I (μW cm^−2^)	k (min^−1^)	I_0_ (μW cm^−2^)	I_1_ (μW cm^−2^)	(I_0_-I_1_) (μW cm^−2^)	Φ
200	0.00815	190	3	187	6.20%
300	0.01317	270	11	259	6.51%
400	0.01714	300	14	286	6.69%
500	0.02005	380	17	363	6.86%
600	0.02440	440	20	420	7.56%
700	0.03002	550	25	525	8.36%
800	0.03386	600	26	574	8.18%

**Table 4:** Transition state parameters for the photo-oxidation of RPE.

I (μW cm^−2^)	Temperature (°C)	∆H^≠^ (kJ mol^−1^)	∆G^≠^ (kJ mol^−1^)	∆S^≠^ (J/(K^−1^ mol^−1^))
200	25	16.695	84.911	−228.915
	30	16.653	86.084	−229.147
	35	16.611	87.200	−229.184
	40	16.570	88.574	−230.045
300	25	15.647	83.707	−228.389
	30	15.605	84.890	−228.662
	35	15.564	85.986	−228.644
	40	15.522	87.152	−228.851
400	25	13.153	83.054	−234.568
	30	13.111	84.145	−234.436
	35	13.070	85.378	−234.766
	40	13.028	86.557	−234.916
500	25	11.989	82.666	−237.170
	30	11.947	83.806	−237.159
	35	11.906	85.060	−237.515
	40	11.864	86.204	−237.508
600	25	11.656	82.179	−236.654
	30	11.615	83.317	−236.641
	35	11.573	84.574	−237.015
	40	11.532	85.708	−236.985
700	25	10.825	81.665	−237.720
	30	10.783	82.849	−237.841
	35	10.742	84.038	−237.975
	40	10.700	85.249	−238.176
800	25	10.126	81.367	−239.063
	30	10.085	82.630	−239.422
	35	10.043	83.780	−239.405
	40	10.002	84.968	−239.510
